# Integrin β1, PDGFRβ, and type II collagen are essential for meniscus regeneration by synovial mesenchymal stem cells in rats

**DOI:** 10.1038/s41598-022-18476-2

**Published:** 2022-08-19

**Authors:** Tsukasa Kitahashi, Ryo Kogawa, Kentaro Nakamura, Ichiro Sekiya

**Affiliations:** 1 Bioscience & Engineering Laboratory, FUJIFILM Corporation, Kanagawa, Japan; 2 Center for Stem Cell and Regenerative Medicine, Tokyo Medical and Dental University, 1-5-45 Yushima, Bunkyo-ku, Tokyo, 113-8510 Japan

**Keywords:** Mesenchymal stem cells, Regeneration, Musculoskeletal system

## Abstract

Synovial mesenchymal stem cells (MSCs) injected into the knee promote meniscus regeneration in several animal models; however, the mode of action is unknown. Our purpose was to identify the molecules responsible for this meniscus regeneration. Rat synovial MSCs were treated with neutralizing antibodies for integrin β1, PDGFRβ, or CD44 or with the CRISPR/Cas9 system to delete *Vcam1*, *Tnfr1*, or *Col2a1* genes. After partial meniscectomy, rat knees were injected with MSCs, and the regenerated meniscus area was quantified three weeks later. The in vivo and in vitro functions were compared between the treated and control MSCs. Anti-integrin β1 neutralizing antibody inhibited in vitro MSC adhesion to collagen-coated chambers, anti-PDGFRβ neutralizing antibody inhibited proliferation in culture dishes, and *Col2a1* deletion inhibited in vitro chondrogenesis. In vivo, the regenerated meniscus area was significantly smaller after injection of MSCs treated with integrin β1 and PDGFRβ neutralizing antibodies or lacking type II collagen gene than after control MSC injection. By contrast, the regenerated areas were similar after injection of control, CD44-, *Vcam1-*, or *Tnfr1* treated MSCs (*n* = 12–16) MSCs. Synovial MSCs injected into the knee joint promoted meniscus regeneration by adhesion to integrin β1 in the meniscectomized region, proliferation by PDGFRβ, and cartilage matrix production from type II collagen.

## Introduction

The meniscus is a fibrocartilaginous structure located between the tibiofemoral articulations^1^. Its roles in the knee are to impart stability, distribute axial loads, promote lubrication of the tibiofemoral joint, and supply nutrients to the articular cartilage^2–5^. Most injured menisci are treated by menisectomy^6^ due to the low healing potential of the meniscus, but meniscectomy leads to the progression of osteoarthritis of the knee^7^. Therefore, the current treatment consensus is to preserve the meniscus^8^. However, meniscus repair is generally possible only for tears along the circumferential fibers that show no degeneration in the first third of the region from the outer edge, where blood supply is abundant^9^. In addition, the rate of revision surgery is higher for meniscus repair than for meniscectomy^10^.

The outcome of meniscus repair can be improved by the injection of mesenchymal stem cells (MSCs)^11^. MSCs can be obtained from various mesenchymal tissues, such as bone marrow and subcutaneous fat, but MSCs derived from the synovium in the knee show high potential for proliferation and chondrogenic differentiation^12^. Furthermore, transplantation of synovial MSCs onto a repaired meniscus can further improve the clinical outcomes of patients with severe meniscus injury^13,14^.

Preclinical studies using anterior medial meniscectomy models in rats^15^, rabbits^16^, pigs^17^, and primates^18^ have shown that injection of synovial MSCs promotes meniscus regeneration. The mechanisms currently proposed to explain how injected synovial MSCs promote meniscus healing include adherence of synovial MSCs to the region around the meniscus defect^17^, MSC proliferation in the joint^15^, and MSC production of cartilage matrix^19^. However, no detailed mode of action has been established. According to previous reports on MSCs, the important molecules include adhesion are integrin β1^20^, VCAM1^21^, and CD44^22^; those involved in proliferation are PDGF^23^ and TNFα^24^ and those involved in matrix production are type II collagen^19,25^. The purpose of this study was to identify the molecules responsible for meniscus regeneration induced by injected MSCs. We diminished or blocked the function of six important related molecules by administering neutralizing antibodies and by knocking out three genes using the Crispr/Cas9 system^26^.

## Results

### Effect of integrin β1 on adhesion and meniscus regeneration in synovial MSCs

**Figure 1:**
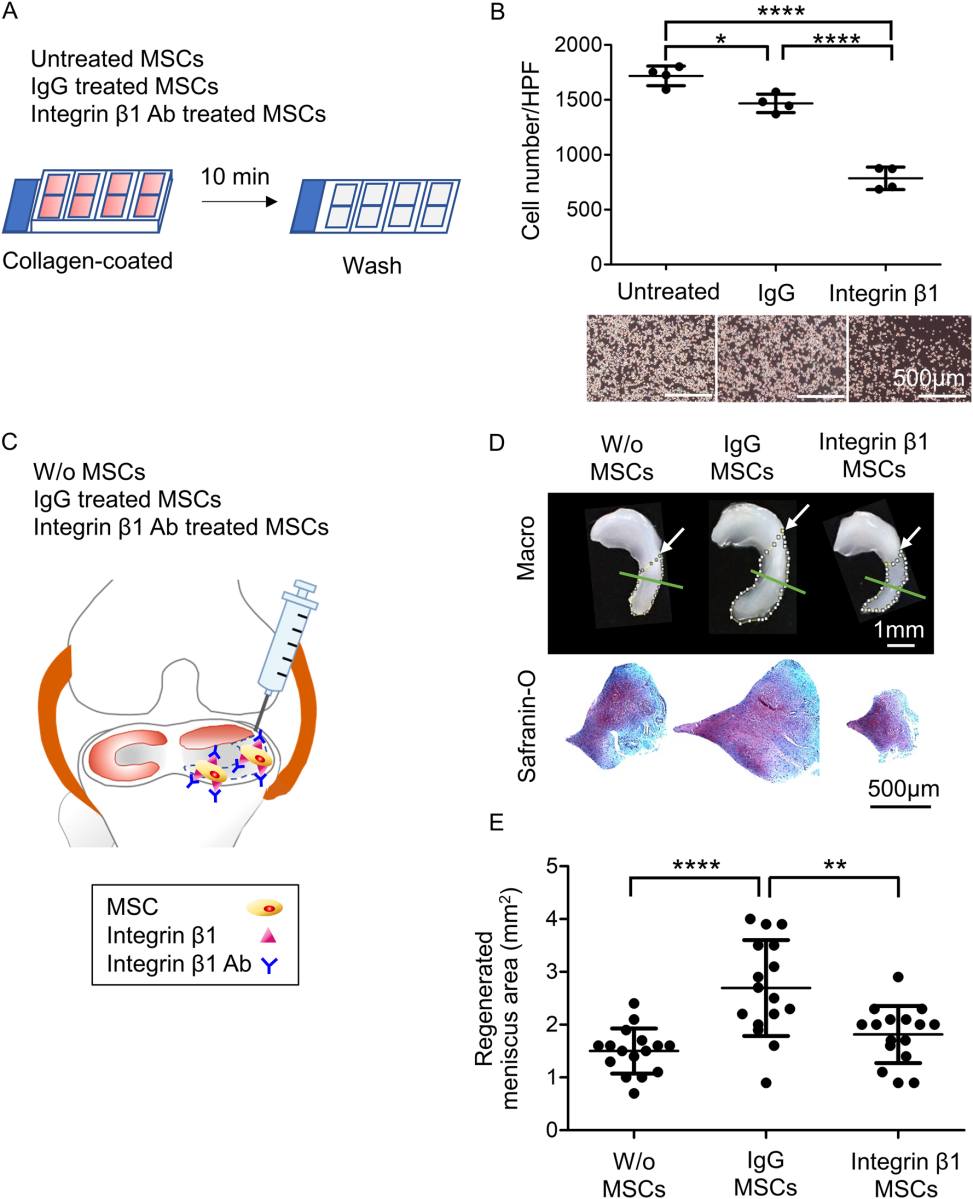
Effect of integrin β1 neutralizing antibody treatment of rat synovial MSCs on adhesion and meniscus regeneration. (**A**) Scheme of the in vitro adhesion experiment. Untreated (control), IgG-treated, and integrin β1 neutralizing antibody–treated MSCs were plated on collagen-coated wells, washed after 10 min, and observed. (**B**) Microscopic images and quantification of adherent cells on collagen-coated slides. Original stock MSCs were used and four wells were tested for each condition. The average values and SD are shown (*n* = 4). HPF, high power field. *, *p* < 0.05; ****, *p* < 0.0001 between each treatment. (**C**) Scheme for in vivo experimental meniscus regeneration. After partial meniscectomy, rat knees were transplanted with vehicle alone (W/o MSCs), or with MSCs treated either with IgG or with integrin β1 neutralizing antibody. Three weeks later, the meniscus was removed and observed macroscopically and microscopically. (**D**) Representative macroscopic images of the meniscus and histological images of the regenerated meniscus by safranin-O staining. The regenerated areas are indicated by the yellow dotted line. Arrow indicates the resected site of the meniscus. Green line indicates the specimen cross section location. (**E**) Quantification of the regenerated area. Original stock MSCs were used, and 16 knees of 8 rats were tested for each condition. The average values and SD are shown (*n* = 16) **, *p* < 0.01; ****, *p* < 0.0001 between each group.

Treatment of MSCs with integrin β1 neutralizing antibody (Fig. 1A) significantly inhibited the adhesion of MSCs to collagen-coated chambers (Fig. 1B). Three weeks after the meniscectomy (Fig. 1C), the injection of only vehicles (W/o MSCs) resulted in a slight increase in the size of the meniscus due to a natural ability to heal (Fig. 1D, Fig. S1). Injection of IgG-treated MSCs significantly increased the size of the meniscus (Fig. 1E), whereas injection of integrin β1-treated MSCs significantly reduced the effect of MSCs on meniscus size. The regions of meniscus regeneration were histologically comparable in all three groups (Fig. 1D, Fig. S2).

In vivo experiments of meniscus regeneration were also performed by manipulating CD44 and *Vcam1*. Meniscus regeneration was equivalent for MSCs treated with CD44 neutralizing antibody or with IgG (Fig. S3). The effect of *Vcam1* gene knockout was comparable to that of wild-type MSCs (Fig. S4).

### Effect of PDGFRβ on proliferation and meniscus regeneration in synovial MSCs

**Figure 2:**
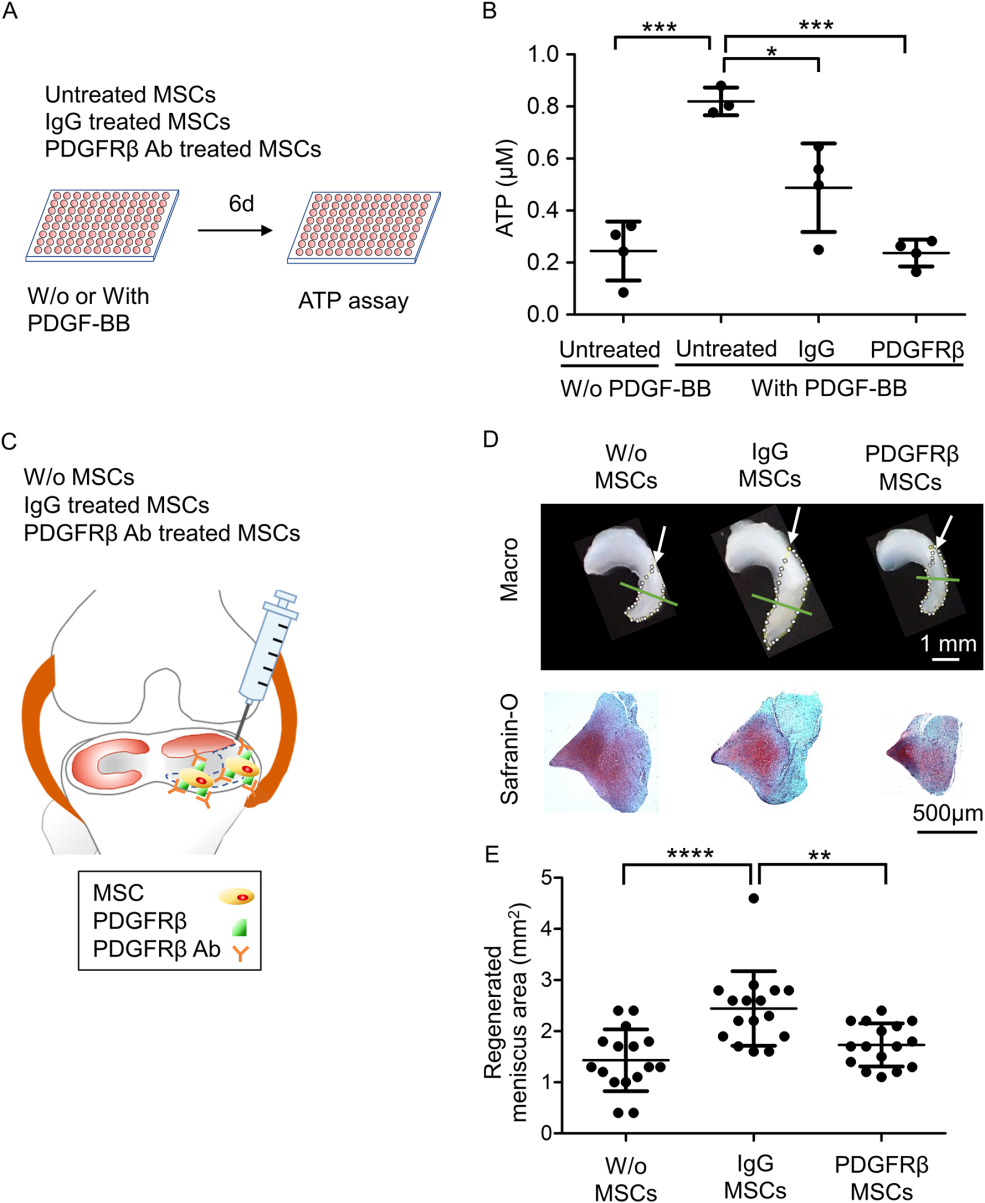
Effect of PDGFRβ neutralizing antibody treatment of rat synovial MSCs on proliferation and meniscus regeneration. (**A**) Scheme of the in vitro proliferation experiment. Untreated, IgG-treated, and PDGFRβ neutralizing antibody–treated MSCs were cultured in well plates with 4 ng/mL PDGF-BB for 6 days. (**B**) Quantification of ATP per well as an indicator of cell number. Original stock MSCs were used and four wells were tested for one condition. The average values and SD are shown (*n* = 4). *, *p* < 0.05; ***, *p* < 0.001 between each treatment. (**C**) Scheme of the in vivo meniscus regeneration experiment. After partial meniscectomy, rat knees were transplanted with vehicle alone (W/o MSCs), IgG-treated MSCs, or PDGFRβ neutralizing antibody–treated MSCs. Three weeks later, the meniscus was removed and observed macroscopically and microscopically. (**D**) Representative macroscopic images of the meniscus and histological images of the regenerated meniscus by safranin-O staining. The regenerated areas are indicated by the yellow dotted line. Arrow indicates the meniscus resected site. Green line indicates the specimen cross section location. (**E**) Quantification of regenerated area. Original stock MSCs were used and 16 knees of 8 rats were tested for one condition. The average values and SD are shown (*n* = 16). **, *p* < 0.01; ****, *p* < 0.0001 between each group.

The ATP assay for MSC proliferation at 6 days (Fig. 2A) showed significantly enhanced proliferation for PDGF-BB (Fig. 2B). Treatment with IgG significantly inhibited proliferation, and anti-PDGFRβ neutralizing antibody treatment further decreased it. Injection of IgG-treated MSCs (Fig. 2C) significantly increased the size of the meniscus (Fig. 2D,E, Fig. S5), whereas injection of PDGFRβ-treated MSCs significantly reduced the effect of MSCs on meniscus size. All three groups showed histological similarities in the regenerated areas of the meniscus (Fig. 2D, Fig. S6).

In vivo experiments of meniscus regeneration using gene-knockout MSCs were also performed for *Tnfr1*. MSCs lacking this gene showed equivalent regeneration of the meniscus to that of the wild-type MSCs (Fig. S7).

### Effect of type II collagen on cartilage matrix synthesis and meniscus regeneration in synovial MSCs

**Figure 3:**
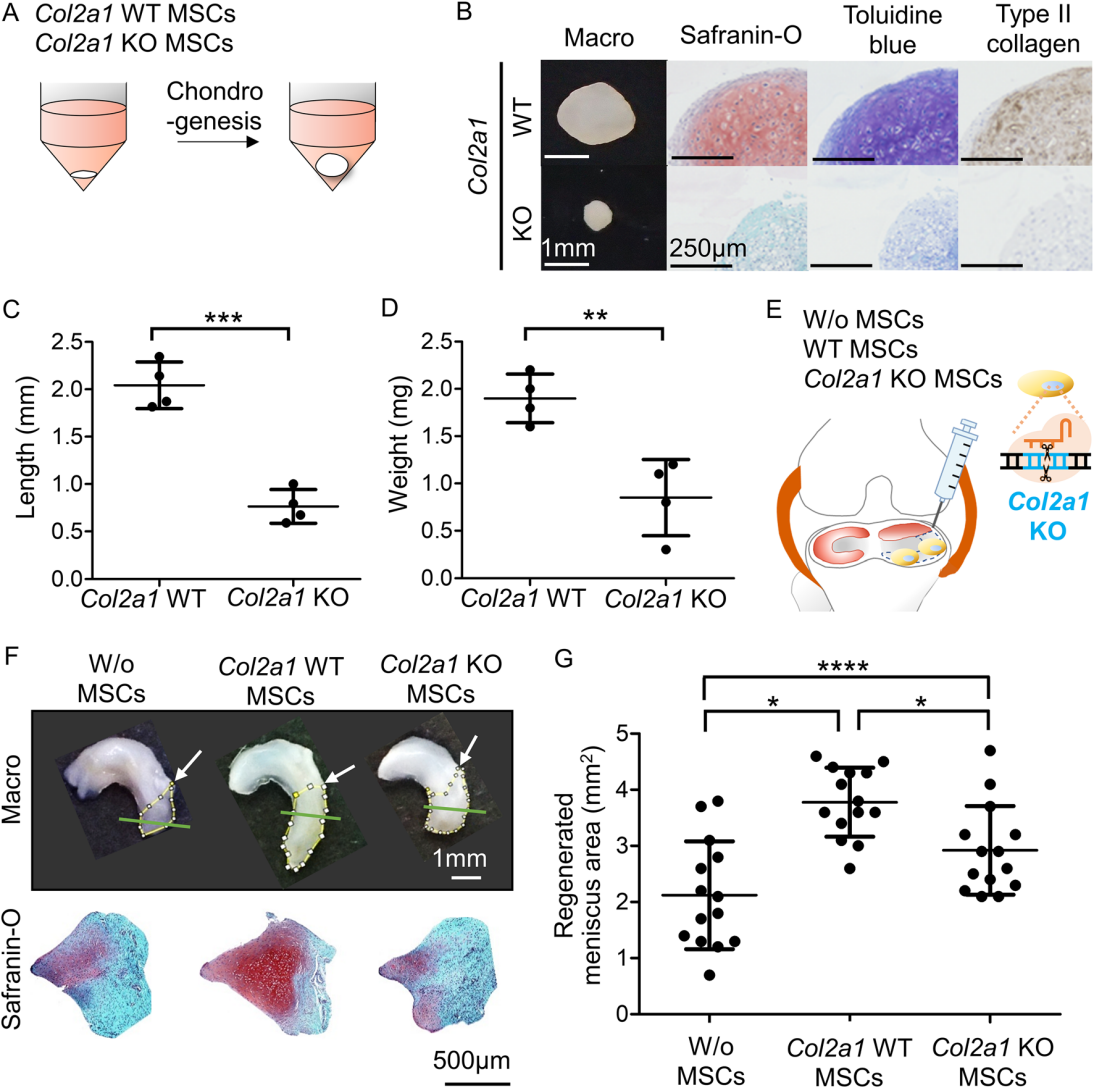
Effect of *Col2a1* gene knockout in rat synovial MSCs on cartilage matrix synthesis and meniscus regeneration. (**A**) Scheme of the in vitro experiment for cartilage matrix synthesis. *Col2a1* wild-type (WT) MSCs and *Col2a1* knockout (KO) MSCs were pelleted and cultured in chondrogenic medium for 21 days. (**B**) Representative macroscopic and histological images of the pellets. (**C**) Length of the pellets. Original stock MSCs were used and four pellets were prepared for one condition. The average values and SD are shown (*n* = 3). ***, *p* < 0.001 between each MSCs. (**D**) Weight of the pellets. The average values and SD are shown (*n* = 3). **, *p* < 0.01; between each MSC type (**E**) Scheme of the in vivo experiment for meniscus regeneration. After partial meniscectomy, rat knees were transplanted with vehicle alone (w/o MSCs), *Col2a1* WT MSCs, or *Col2a1* KO MSCs. Three weeks later, the meniscus was removed and observed macroscopically and microscopically. (**F**) Representative macroscopic images of the meniscus and histological images of the regenerated area by safranin-O staining. The regenerated areas are indicated by the yellow dotted line. Arrow indicates the meniscus resected site. Green line indicates the specimen cross section location. (**G**) Quantification of the regenerated area. Original stock MSCs were used and 14 knees of 7 rats were tested for one condition. The average values and SD are shown (*n* = 14). *, *p* < 0.05; ****, *p* < 0.0001 between each group.

The results of the in vitro chondrogenesis assay carried out for 21 days (Fig. 3A) showed an inhibition of cartilage matrix synthesis, including type II collagen expression, in *Col2a1*-knockout MSCs (Fig. 3B). The cell pellet was significantly smaller and lighter in *Col2a1* knockout MSCs than in *Col2a1* wild-type MSCs (Fig. 3C,D ). The in vivo experiment for meniscus regeneration (Fig. 3E) also showed a significantly smaller regenerated meniscus area following treatment with *Col2a1* knockout MSCs than with *Col2a1* wild-type MSCs, but this area was significantly larger following treatment with *Col2a1* knockout MSCs than with vehicle-treated MSCs (W/o MSCs) (Fig. 3F,G , Fig. S8). No obvious histological difference was detected in the regenerated area of the meniscus in the three groups (Fig. 3D, Fig. S9).

## Discussion

Transplantation of synovial MSCs has been shown to promote meniscus regeneration^11,13–18^, but the molecules and signals that are essential for this process remain obscure. Previous regeneration studies of MSCs injected into tissues other than meniscus and articular cartilage have identified important molecules and signals, including integrin β1^20,27^, VCAM1^21^, and CD44^22^ for adhesion, PDGF^23^ and TNFα^24^ for proliferation, and type II collagen^19,25^ for matrix synthesis. In this study, we investigated whether these molecules are essential for the promotion of meniscus regeneration by synovial MSCs, both in vitro and in vivo.

Integrins are representative molecules associated with cell adhesion and with a superfamily of cell adhesion receptors^27^ that form αβ heterodimers and adhere to various extracellular matrices^27^. Among them, integrin β1 forms heterodimers with most integrin α subunits and plays a role in adhesion to various extracellular matrices^27^. Our findings showed that inhibition of integrin β1 by a neutralizing antibody inhibited the adhesion of synovial MSCs to collagen-coated chambers and impaired meniscus regeneration after transplantation of synovial MSCs. Horie et al*.* reported that synovial MSCs injected into the knees of rats with a partially resected meniscus adhered to the dissected edge of the meniscus^15^. These findings suggest that inhibition of integrin β1 prevents meniscal regeneration by interfering with the adhesion of synovial MSCs to the meniscus lesion.

CD44 and VCAM1 are also molecules involved in cell adhesion^21,22^, but neither anti-CD44 neutralizing antibody treatment nor *Vcam1* gene knockout decreased the ability of MSCs to regenerate the meniscus. MSCs also express other adhesion molecules, including CD168^28^, ICAM1^21^, and ALCAM^21^, which have similar functions to CD44 and VCAM1 for substrate adhesion. We speculate that these molecules may compensate for the adhesive function of CD44 or VCAM1 when they are absent in synovial MSCs.

PDGF promotes the proliferation of synovial MSCs in cell culture systems^23^. Receptors for PDGF form homodimers or heterodimers, and since synovial MSCs express PDGFRβ most strongly among the receptors^29^, we focused on PDGFRβ. Treatment of PDGFRβ with neutralizing antibodies inhibited PDGF-induced cell proliferation in vitro, and injection of cells treated with PDGFRβ-neutralizing antibodies prevented meniscus regeneration in vivo. Horie et al*.* reported that intra-articular injection of luciferase-expressing synovial MSCs into rats after meniscectomy increased the luminescence intensity approximately threefold after 3 days^15^. These results suggest that inhibition of PDGFRβ function inhibits the proliferation of synovial MSCs attached to the injured meniscus, resulting in impairment of meniscus regeneration.

TNFα also promotes proliferation of synovial MSCs in a cell culture system^24^. The cellular response to TNFα is induced via two receptors, TNFR1 and TNFR2. TNFR1 is expressed in most tissues, whereas TNFR2 is mainly localized on immune cells^30^; therefore, we focused on TNFR1. Synovial MSCs lacking TNFR1 did not affect meniscus regeneration in vivo, indicating that TNFα signaling was not essential for the effects of synovial MSCs on meniscus regeneration.

Collagen, which comprises about 20% of the meniscus, is the second most abundant component after water^31^. *Col2a1* is a representative gene that encodes type II collagen in the meniscus^32^. In the present study, *Col2a1* knockout in synovial MSCs eliminated positive cartilage matrix staining for safranin-O or toluidine blue in vitro, and the produced cartilage pellets were smaller in size for the *Col2a1* knockout MSCs than for the controls. In vivo studies demonstrated that *Col2a1* knockout in synovial MSCs suppressed meniscus regeneration. Decreased expression of type II collagen due to mutation in *Col2a1* has been reported in patients with genetic disorders of cartilage^33^. Chen et al*.* also reported that the addition of type II collagen protein promoted the production of cartilage matrix in MSCs^34^. The association between type II collagen expression and meniscus formation remains to be fully elucidated; however, these findings show that the *Col2a1* gene is essential for the chondrogenic differentiation of synovial MSCs in vitro and for meniscus regeneration in vivo.

We have identified essential molecules in synovial MSCs for meniscus regeneration. Generally, the mode of action is less clear for MSCs administered systemically or intraarticularly than for small molecular compounds and antibody drugs, and items for assessing the quality of cell therapy products are often restricted to viability, surface antigens, and in vitro differentiation potential. The molecules identified as essential in this study can serve as quality items and markers for elucidating the efficacy of synovial MSCs destined for use in meniscus regeneration therapy.

Approximately 4 elapsed from the time the cells were prepared to the time the cells were injected, and the cells were kept on ice throughout this interval. This may have affected cell viability and activity. However, the cells remaining after completion of the injections to all rats did not show any decreases in viability. Furthermore, no association was noted between the order of the injection and the regenerated meniscus area in rats. These observations indicate that storage of the cells on ice for 4 h did not significantly affect cell viability or activity.

The regenerated meniscus area was used as a measure of the effect of the injected cells. A similar approach has been reported in studies using rats^15^, rabbits^16^, and pigs^17,35^. In those studies, the area of the regenerated meniscus correlated with the stainability with safranin O and type 2 collagen. It also correlated with the degree of inhibition of degeneration of the articular cartilage adjacent to the meniscus. Furthermore, in pigs, it was correlated with the tensile strength to failure of the meniscus^35^. The area of the regenerated meniscus area is a reliable indicator for determine the effect of the injected cells.

This study had several limitations. One was that the in vitro analysis for integrin β1 and PDGFRβ does not reflect the in vivo biological events, so in vivo analyses of adhesion and cell proliferation are still required. Another limitation was that the regenerated meniscus was not quantitatively evaluated in histological tissue sections. However, none of the histological images shown in the Supplementary Figures show any marked differences among the groups. A third limitation was that we did not conduct histological examinations for molecules that did not cause any obvious size differences in the regenerated meniscus area. Had we conducted these examinations, we might have detected some differences. A fourth limitation was that no validation assays were performed for the anti-CD44 antibody, so, the problem of neutralizing action could not be completely ruled out. However, we used antibodies whose blocking action was guaranteed by the manufacturer. A further limitation was that we did not analyze where the injected MSCs would be engrafted in the joint or how long they would remain there. In our previous study using the same rat model, the injected cells accumulated at the site of meniscectomy after 1 day and were still detectable in the regenerating meniscus after 12 weeks, and were expressing type 2 collagen^15^. A last limitation was that we used rat cells and examined the MSCs in rat knees. Although rat and human MSCs have many properties in common, further study is needed to confirm that the results in humans will be similar to those obtained in rats.

**Figure 4:**
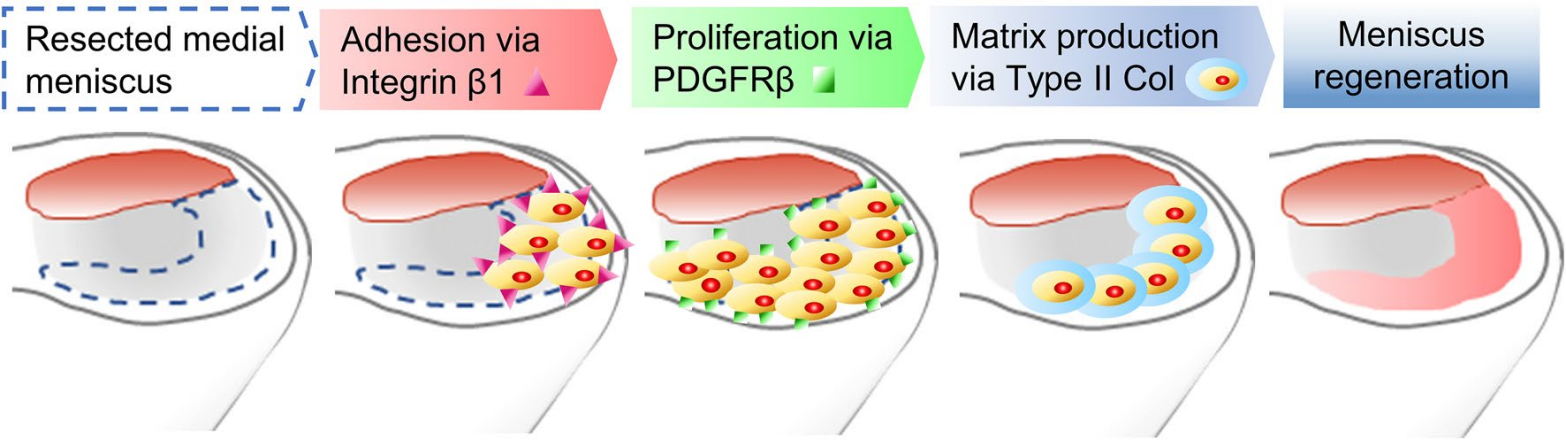
Summary. Synovial MSCs injected into the knee joint adhered to the area around the meniscectomized region via integrin β1, proliferated via PDGFRβ, produced cartilage matrix by type II collagen, and promoted meniscus regeneration.

The mechanism by which intra-articular injection of synovial MSCs promotes meniscus regeneration can be divided into three steps: adhesion of the MSCs to the area around the meniscus defect, MSC proliferation in the joint, and MSC production of cartilage matrix. In this study, we identified essential molecules for each of these steps. Synovial MSCs injected into the knee joint adhered to the area around the meniscectomized region via integrin β1, proliferated via PDGFRβ, produced cartilage matrix through type II collagen, and promoted meniscus regeneration (Fig. 4). Conversely, CD44, VCAM1, and TNFR1, which were molecules predicted to be important at the start of this study, were deemed nonessential for meniscus regeneration.

## Methods

### Animals

All animal care and experiments were conducted in accordance with the ARRIVE (Animal Research: Reporting of In Vivo Experiments) guidelines and the institutional guidelines of the Animal Committee that includes the first author as a member. Male/female Lewis rats (6–10 weeks old) were purchased from The Jackson Laboratory Japan, Inc. (Yokohama, Japan) (total of 130 animals). All rats were freely allowed access to food, water, and activity. The rats were maintained under a 12 h dark–light cycle at controlled temperature (20–26 °C) and humidity (30–70%).

### Isolation of rat synovial MSC

Male rats were euthanized by bleeding from the inferior vena cava under isoflurane anesthesia. Synovium was harvested from the infrapatellar fat pad in both knees of 10 rats, pooled together, and minced. The minced synovium was then digested with 0.3% Collagenase V solution (Merck KGaA, Darmstadt, Germany) in a water bath at 37 °C for 2 h. The cells were cultured in α-minimum essential medium (αMEM; Thermo Fisher Scientific, Waltham, MA, USA) with 20% fetal bovine serum (FBS; Thermo Fisher Scientific, MA, USA) for 8 days under conditions of 5% CO_2_ and 37 °C. The cells were then harvested, prepared as the original stocks, and cryopreserved in a freezer (CLN-1700CWE, Nihon Freezer, Tokyo, Japan) at − 150 °C. For use, synovial MSCs from the original stocks were cultured for 7 days and harvested, and combined. The cells were used for further analyses without any sorting. The cells showed similar surface antigens and multidifferentiation abilities to those of MSCs, as reported previously^36^.

### Neutralizing antibody–treated MSCs

The neutralizing antibodies were anti-integrin β1 antibody (purified anti-mouse/rat CD29 antibody; BioLegend Inc., San Diego, CA, USA), anti-PDGF Receptor β antibody (R&D Systems Inc., Minneapolis, MN, USA), and anti-CD44 antibody (Absolute Antibody Ltd., Cleveland, UK). The controls were Purified Armenian Hamster IgG Isotype Ctrl Antibody (BioLegend Inc.), Normal Goat IgG Control (R&D Systems Inc.), and IgG Isotype Ctrl (Thermo Fisher Scientific, MA, USA), respectively. Synovial MSCs at 5 × 10^6^ cells were suspended in 900 μL of PBS with 2% FBS for neutralizing integrin β1 or 450 μL of PBS with 2% FBS for neutralizing PDGFRβ. A 50 μg sample of neutralizing antibodies for integrin β1 and CD44, and 100 μg for PDGFRβ were added to the suspensions, and the cells were allowed to react for 1 h on ice, washed, and corrected. The final volume was then adjusted with vehicle to give a concentration of 5 × 10^6^ cells/50 μL.

### Cell adhesion assay

Untreated MSCs, IgG-treated MSCs, and integrin β1 neutralizing antibody–treated MSCs of 1 × 10^5^ were suspended in 10 µL of PBS with 10 mM MgCl_2_ and placed for 10 min on 8-well culture slides coated with type I collagen (Corning Inc., Corning, NY, USA). After washing the slides with PBS containing 10 mM MgCl_2_, the number of cells in one high power field (HPF) was counted^20^.

### Cell proliferation assay

Untreated MSCs, IgG-treated MSCs, and PDGFRβ neutralizing antibody–treated MSCs were plated at 1 × 10^3^ cells/100 μL in complete medium containing 20% FBS in 96-well plates (Corning). On the next day, the medium was replaced with 100 μL of αMEM supplemented with 0.5% FBS, and 4 ng/mL of PDGF-BB (R&D Systems Inc.). Untreated MSCs were also cultured in αMEM supplemented with 0.5% FBS but without PDGF-BB. These MSCs in the four experimental conditions were cultured at 37 °C for 6 days^37^. The cell proliferation rate was determined by an ATP assay using Cell Titer Glo (Promega Corp., Madison, WI, USA) according to manufacturer’s protocol.

### Knockout of *Col2a1*, *Vcam1*, and *Tnfr1* genes in synovial MSCs

Synovial MSCs from the original stock were cultured in 24-well plates (Corning Inc.) at a density of 8 × 10^4^ cells/well in 500 μL of the complete medium for 1 day. For Cas9 protein transfection, 50 μL of the mixture containing TrueCut Cas9 Protein v2 (Invitrogen, cat# A36498), Lipofectamine CRISPRMAX Cas9 Transfection Reagent (Thermo Fisher Scientific), Lipofectamine Cas9 Plus Reagent (Thermo Fisher Scientific), Opti-MEM I Reduced Serum Medium (Thermo Fisher Scientific), and 240 ng of modified custom TruGuide sgRNA (Thermo Fisher Scientific) for the *Col2a1, Vcam1*, and *Tnfr1* genes (Table S1) were added and the plates were incubated for 2 days. Target gene editing was confirmed by extracting DNA from 1 × 10^5^ cells, amplifying the target gene by PCR, and detecting the mutant gene using the GenArt Genomic Cleavage Detection Kit (Thermo Fisher Scientific). For cell cloning, the cells with mutant genes were collected, plated in 96-well plates at a concentration of 0.5 cells/well, and cultured to provide over 10^8^ cells for transplantation. To confirm gene knockout, DNA was extracted from 1 × 10^5^ cloned cells, and its sequences were confirmed by Sanger sequencing (Fig. S10, Table S2–S4).

### In vitro chondrogenesis assay

In a 15 mL polypropylene tube, 2.5 × 10^5^ synovial MSCs were suspended in 1 mL of chondrogenic media consisting of DMEM high glucose (Thermo Fisher Scientific), including 10 ng/mL TGF-β3 (R&D Systems Inc.), 3.92 μg/mL dexamethasone (FUJIFILM Wako Pure Chemical Corp., Osaka, Japan), 50 μg/mL L-ascorbic acid 2-phosphate (Cayman Chemical Company, Ann Arbor, MI, USA), 40 μg/mL L-proline (MP Biomedicals, Irvine, CA, USA), 1 μg/mL sodium pyruvate (Thermo Fisher Scientific), 1% ITS-X supplement (× 100) (FUJIFILM Wako Pure Chemical Corp.), and 0.5 μg/mL BMP-2 (R&D Systems Inc.). The cells were pelleted by centrifugation at 450 g for 10 min. The pellets were cultured for 3 weeks, with the media changed every 3–4 days^12,19,29^. For histological analysis, the pellets were embedded in paraffin, cut into 5 μm thick sections, stained with safranin-O and toluidine blue, and immunostained for type II collagen using the purified Anti-hCL(II) IgG antibody (KYOWA PHARMA CHEMICAL CO., LTD., Toyama, Japan)^15,38^.

### Meniscectomy and synovial MSC transplantation

Female rats were anesthetized with isoflurane. Both the right and left knee joints underwent surgery. A medial parapatellar incision and lateral dislocation of the patellar tendon were conducted to expose the medial meniscus. The anterior insertional ligament of the medial meniscus was transected to dislocate the medial meniscus anteriorly, and the medial meniscus was resected at the level of the medial collateral ligament. The capsule was closed using nylon sutures, and MSCs at 5 × 10^6^ cells/50 µL or vehicle were injected into the knee joint using a 28G needle. (All MSCs and vehicle were stored on ice until administration). The knee joint was moved three times, and the skin was sutured^15^. Surgical treatment and administration were performed together per condition instead of randomly, in order of cell preparation. The time required was approximately 40 min per animal for both knees. The rats were allowed to walk freely in their cages after the surgery. Eight rats per condition were used for Integrin β1 and PDGFRβ experiments, 6 or 7 for CD44 experiments, and 7 for each of the *Vcam1* KO, *Tnfr1* KO and *Col2a1* KO experiments.

### Evaluation of the regenerated meniscus

Three weeks after the MSC injection, the knee joint was removed, and the medial meniscus was photographed. The regenerated area of the meniscus was measured with Image J 1.53e (National Institutes of Health, Bethesda, MD, USA). The meniscus was immersed in a 10% neutral buffered formalin solution and decalcified with 0.5% EDTA (pH 7.5) for 3 days at 4 °C, followed by gradient replacement with 20% sucrose for 24 h at 4 °C. The center of the regenerated area of the meniscus was radially sectioned and histologically observed with safranin-O staining.

### Statistical analysis

Comparisons between the two groups were performed using an unpaired t-test, and comparisons among the three or more groups were performed using one-way ANOVA with Bonferroni's Multiple Comparison Test. Statistical tests were conducted using GraphPad Prism ver. 5.04 (GraphPad Software Inc., San Diego, CA, USA). A *P* value of < 0.05 was considered statistically significant. *, *p* < 0.05; **, *p* < 0.01; ***, *p* < 0.001; ****, *p* < 0.0001.

### Ethical approval

All experimental protocols and studies were approved by the Animal Care and Use Committee (reference number: A-1–200,136, A-1–210,027, A-1–210,031, A-1–210,033, A-1–210,069, A-1–210,072) of FUJIFILM Corporation. All animal care and experiments were performed in accordance with the institutional guidelines of the Animal Committee of FUJIFILM Corporation.

## Supplementary Information

Supplementary Information 1.

Supplementary Information 2.

Supplementary Information 3.

Supplementary Information 4.

Supplementary Information 5.

Supplementary Information 6.

Supplementary Information 7.

Supplementary Information 8.

Supplementary Information 9.

Supplementary Information 10.

Supplementary Information 11.

## Data Availability

The data sets obtained and analyzed in the current study are available from the corresponding author on reasonable request.
