# Supplementary material for: Integrin β1, PDGFRβ, and type II collagen are essential for meniscus regeneration by synovial mesenchymal stem cells in rats
Source: Sci Rep. 2022 Aug 19;12:14148. doi: 10.1038/s41598-022-18476-2 (PMC9391488; doi:10.1038/s41598-022-18476-2)
Supplement: Supplementary file 1 — Supplementary Information 1. [file 41598_2022_18476_MOESM1_ESM.docx]

**Supplementary Table**

**Table S1.** Guide RNA sequence.

| Target gene | sgRNA |
| --- | --- |
| *Vcam1* | GACAGACAGTCCCCTCAATGGGG |
| *Tnfr1* | CCCATCGTGCCTGGCCTGCTGCT |
| *Col2a1* | CCACGGTCCTACAATGTCAGGGC |

**Table S2.** Amino acid sequence of COL2A1 gene mutations in synovial MSCs. There were two types of mutations.

| COL2A1 WT | COL2A1 KO |
| --- | --- |
| MIRLGAPQSLVLLTLLIATVLQCQGQDARKLGPKGQKGEPGDIKDIIGPKGPPGPQGPAGEQGPRGDRGDKGERGAPGPRGRDGEPGTPGNPGPPGPPGPPGPPGLGGGNFAAQMAGGFDEKAGGAQMGVMQGPMGPMGPRGPPGPAGAPGPQGFQGNPGEPGEPGVSGPIGPRGPPGPAGKPGDDGEAGKPGKAGERGLPGPQGARGFPGTPGLPGVKGHRGYPGLDGAKGEAGAPGVKGESGSPGENGSPGPMGPRGLPGERGRTGPAGAAGARGNDGQPGPAGPPGPVGPAGGPGFLGAPGAKGEAGPTGARGPEGAQGSRGEPGNPGSPGPAGASGNPGTDGIPGAKGSAGAPGIAGAPGFPGPRGPPGPQGATGPLGPKGQTGEPGIAGFKGEQGPKGETGPAGPQGAPGPAGEEGKRGARGEPGGAGPIGPPGERGAPGNRGFPGQDGLAGPKGAPGERGPSGLAGPKGANGDPGRPGEPGLPGARGLTGRPGDAGPQGKVGPSGAPGEDGRPGPPGPQGARGQPGVMGFPGPKGANGEPGKAGEKGLAGAPGLRGLPGKDGETGAAGPPGPSGPAGERGEQGAPGPSGFQGLPGPPGPPGEGGKQGDQGIPGEAGAPGLVGPRGERGFPGERGSPGAQGLQGPRGLPGTPGTDGPKGAAGPDGPPGAQGPPGLQGMPGERGAAGIAGPKGDRGDVGEKGPEGAPGKDGGRGLTGPIGPPGPAGANGEKGEVGPPGPSGSTGARGAPGERGETGPPGPAGFAGPPGADGQPGAKGDQGEAGQKGDAGAPGPQGPSGAPGPQGPTGVTGPKGARGAQGPPGATGFPGAAGRVGPPGSNGNPGPAGPPGPAGKDGPKGARGDTGAPGRAGDPGLQGPAGAPGEKGEPGDDGPSGSDGPPGPQGLAGQRGIVGLPGQRGERGFPGLPGPSGEPGKQGAPGASGDRGPPGPVGPPGLTGPAGEPGREGSPGADGPPGRDGAAGVKGDRGETGALGAPGAPGPPGSPGPAGPTGKQGDRGEAGAQGPMGPSGPAGARGIAGPQGPRGDKGEAGEPGERGLKGHRGFTGLQGLPGPPGPSGDQGTSGPAGPSGPRGPPGPVGPSGKDGSNGIPGPIGPPGPRGRSGETGPAGPPGNPGPPGPPGPPGPGIDMSAFAGLGQREKGPDPLQYMRADEADSTLRQHDVEVDATLKSLNNQIESIRSPDGSRKNPARTCQDLKLCHPEWKSGDYWIDPNQGCTLDAMKVFCNMETGESCVYPNPATVPRKNWWSSKSKEKKHIWFGETMNGGFHFSYGDGNLAPNTANVQMTFLRLLSTEGSQNITYHCKNSIAYLDEAAGNLKKALLIQGSNDVEMRAEGNSRFTYTALKDGCTKHTGKWGKTIIEYRSQKTSRLPIVDIAPMDIGGPDQEFGVDIGPVCFL* | MIRLGAPQSLVLLTLLIATXPTMSGPGCPKIRAKGAERRTWRYQRYHRT* |
|  | MIRLGAPQSLVLLTLLIAYNVRARMPEN* |

*, Stop codon; underlined, mutant amino acid sequences.

**Table S3.** Amino acid sequence of VCAM1 gene mutations in synovial MSCs.

| VCAM1 WT | VCAM1 KO |
| --- | --- |
| MPVKMVAIFGASTVLWILFAVSQAFKIEISPEYKTLAQIGDSMLLTCSTTGCESPSFSWRTQIDSPLNGKVKTEGAKSVLTMDPVSFENEHSYLCTATCNSGKLERGIQVDIYSFPKDPEIQFSGPLEVGKPVMVKCLAPDVYPIDRLEIELFKGDRLMKKQDFVDEMAKKSLETKSLEVIFTPVIEDIEKALVCRAKLYIDQTDSIPKERETVRELQVYTSPKNTEISVHPSTRLHEGAAVTMTCASEGLPAPEIFWSKKLDNGVLQLLSGNATLTLIAMRMEDSGIYVCEGVNLVGRDKTEVELIVQEKPFTVDISPGSQVAAQVGDSVVLTCAAVGCDSPSFSWRTQTDSPLNGEVRDEGATSTLTLSPVGVEDEHSYLCTVTCQRRKLEKTIQVEVYSFPEDPEIEISGPLVHGRPVTVNCTVPNVYPFDHLEIELLKGETTLLNKFLREEIGTKSLETKSLEMTFIPTAEDTGKALVCLAKLHSSQMESEPKQRQSTQTLYVNVAPKEPTIWVSPSPVPEEGSPVNLTCSSDGFPTPKILWSRQLKNGELQPLSQNTTLSFMATKMEDSGIYVCEGINEAGISKKSVELIIQGSSKDIQLTVFPSKSVKEGDTVIISCTCGSVPEIWIILKKKAKTGDMVLKSVNGSYTIRKAQLQDAGVYECESKTEVGSQLRSLTLDVKGKENNKDYFSPELLALYFASSLVIPAIGMIIYFARKANMKGSYSLVEAQKSKV* | MPVKMVAIFGASTVLWILFAVSQAFKIEISPEYKTLAQIGDSMLLTCSTTGCESPSFSWRTQIDSPLNGKVKTEGAKSVLTMDPVSFENEHSYLCTATCNSGKLERGIQVDIYSFPKDPEIQFSGPLEVGKPVMVKCLAPDVYPIDRLEIELFKGDRLMKKQDFVDEMAKKSLETKSLEVIFTPVIEDIEKALVCRAKLYIDQTDSIPKERETVRELQVYTSPKNTEISVHPSTRLHEGAAVTMTCASEGLPAPEIFWSKKLDNGVLQLLSGNATLTLIAMRMEDSGIYVCEGVNLVGRDKTEVELIVQEKPFTVDISPGSQVAAQVGDSVVLTCAAVGCDSPSFSWRTQTDSEG* |

*, Stop codon; underlined, mutant amino acid sequences.

**Table S4.** Amino acid sequence of TNFR1 gene mutations in synovial MSCs.

| TNFR1 WT | TNFR1 KO |
| --- | --- |
| MGLPIVPGLLLSLVLLALLMGIHPSGVTGLVPSLGDREKRDNLCPQGKYAHPKNNSICCTKCHKGTYLVSDCPSPGQETVCEVCDKGTFTASQNHVRQCLSCKTCRKEMFQVEISPCKADMDTVCGCKKNQFQRYLSETHFQCVDCSPCFNGTVTIPCKEKQNTVCNCHAGFFLSGNECTPCSHCKKNQECMKLCLPPVANVTNPQDSGTAVLLPLVIFLGLCLLFFICISLLCRYPQWRPRVYSIICRDSAPVKEVEGEGIVTKPLTPASIPAFSPNPGFNPTLGFSTTPRFSHPVSSTPISPVFGPSNWHNFVPPVREVVPTQGADPLLYGSLNPVPIPAPVRKWEDVVAAQPQRLDTADPAMLYAVVDGVPPTRWKEFMRLLGLSEHEIERLELQNGRCLREAHYSMLEAWRRRTPRHEATLDVVGRVLCDMNLRGCLENIRETLESPAHSSTTHLPR* | MGLPICLACCCHWCSWLC* |

*, Stop codon; underlined, mutant amino acid sequences.
