# Supplementary figures and images for: Integrin β1, PDGFRβ, and type II collagen are essential for meniscus regeneration by synovial mesenchymal stem cells in rats
Source: Sci Rep. 2022 Aug 19;12:14148. doi: 10.1038/s41598-022-18476-2 (PMC9391488; doi:10.1038/s41598-022-18476-2)

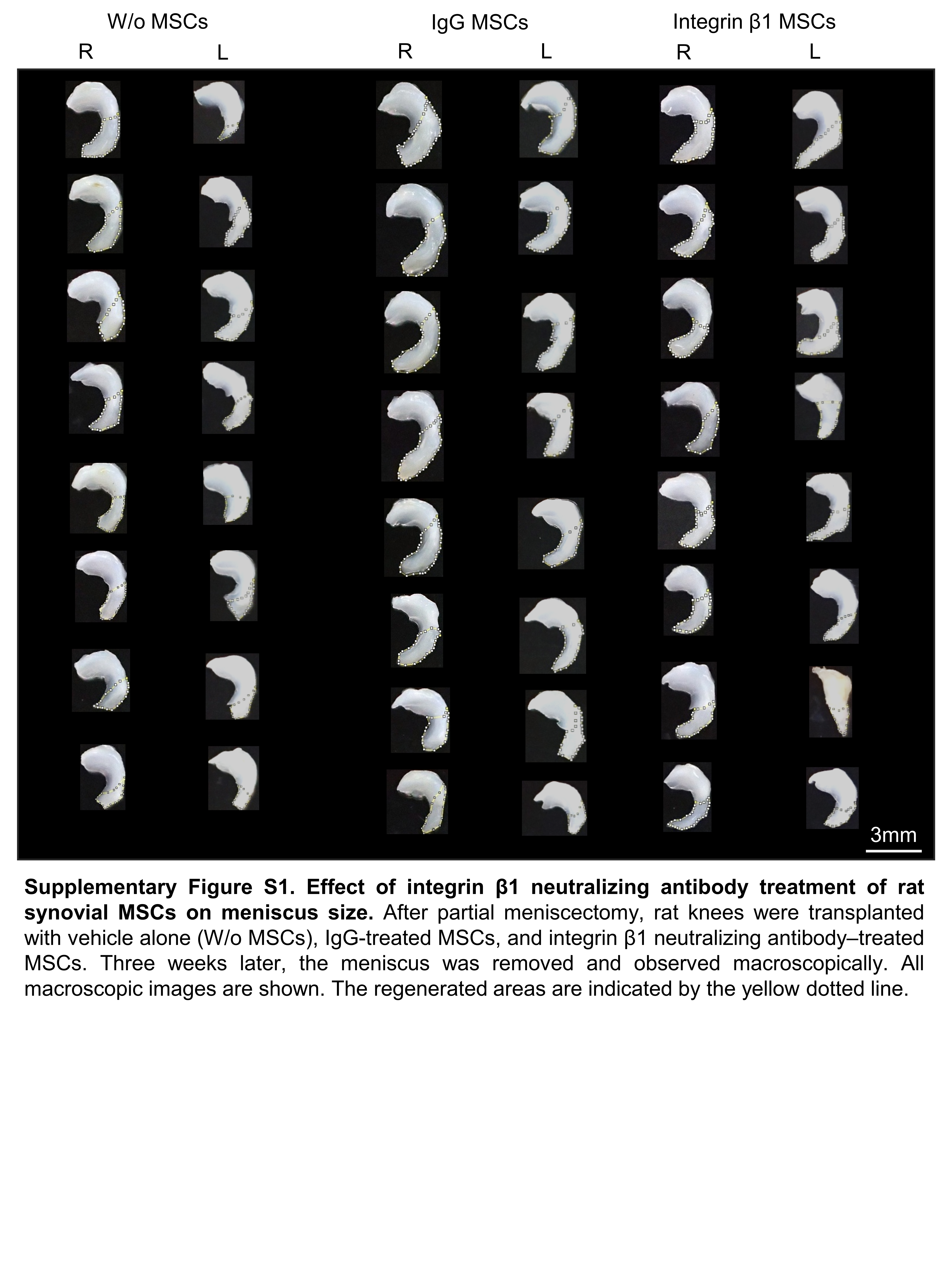

Supplement: Supplementary file 2 — Supplementary Information 2. [file 41598_2022_18476_MOESM2_ESM.tif]

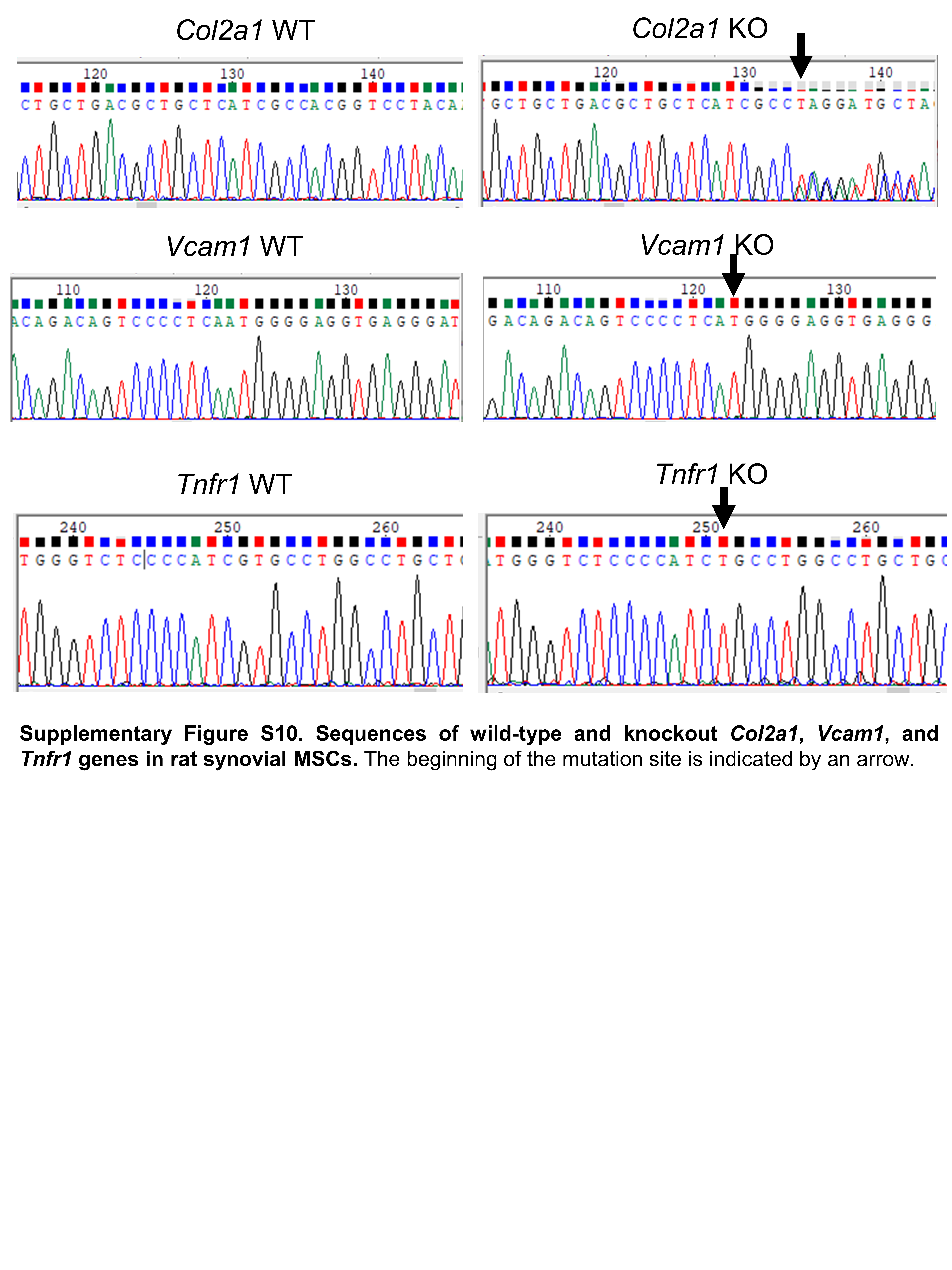

Supplement: Supplementary file 3 — Supplementary Information 3. [file 41598_2022_18476_MOESM3_ESM.tif]

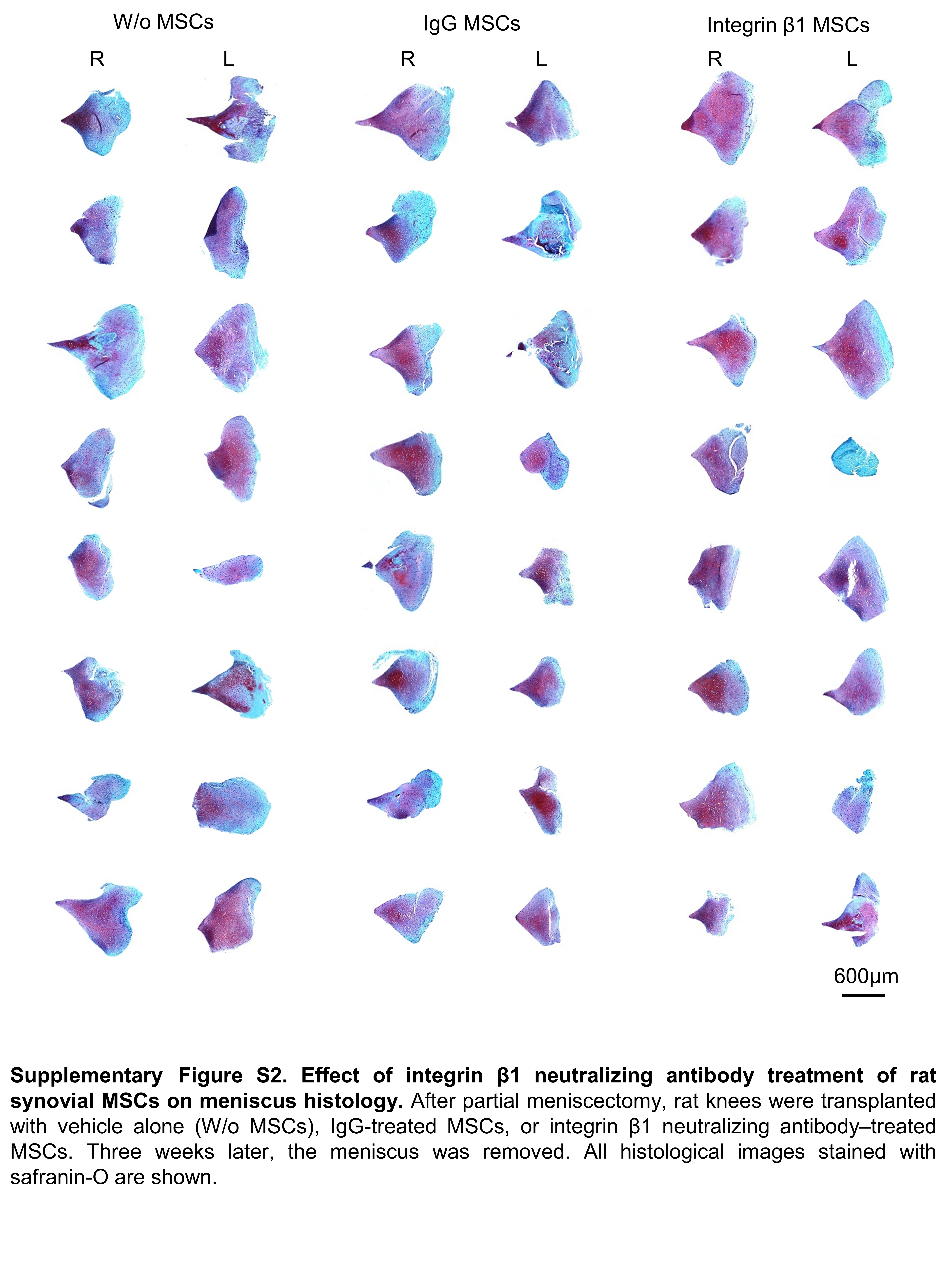

Supplement: Supplementary file 4 — Supplementary Information 4. [file 41598_2022_18476_MOESM4_ESM.tif]

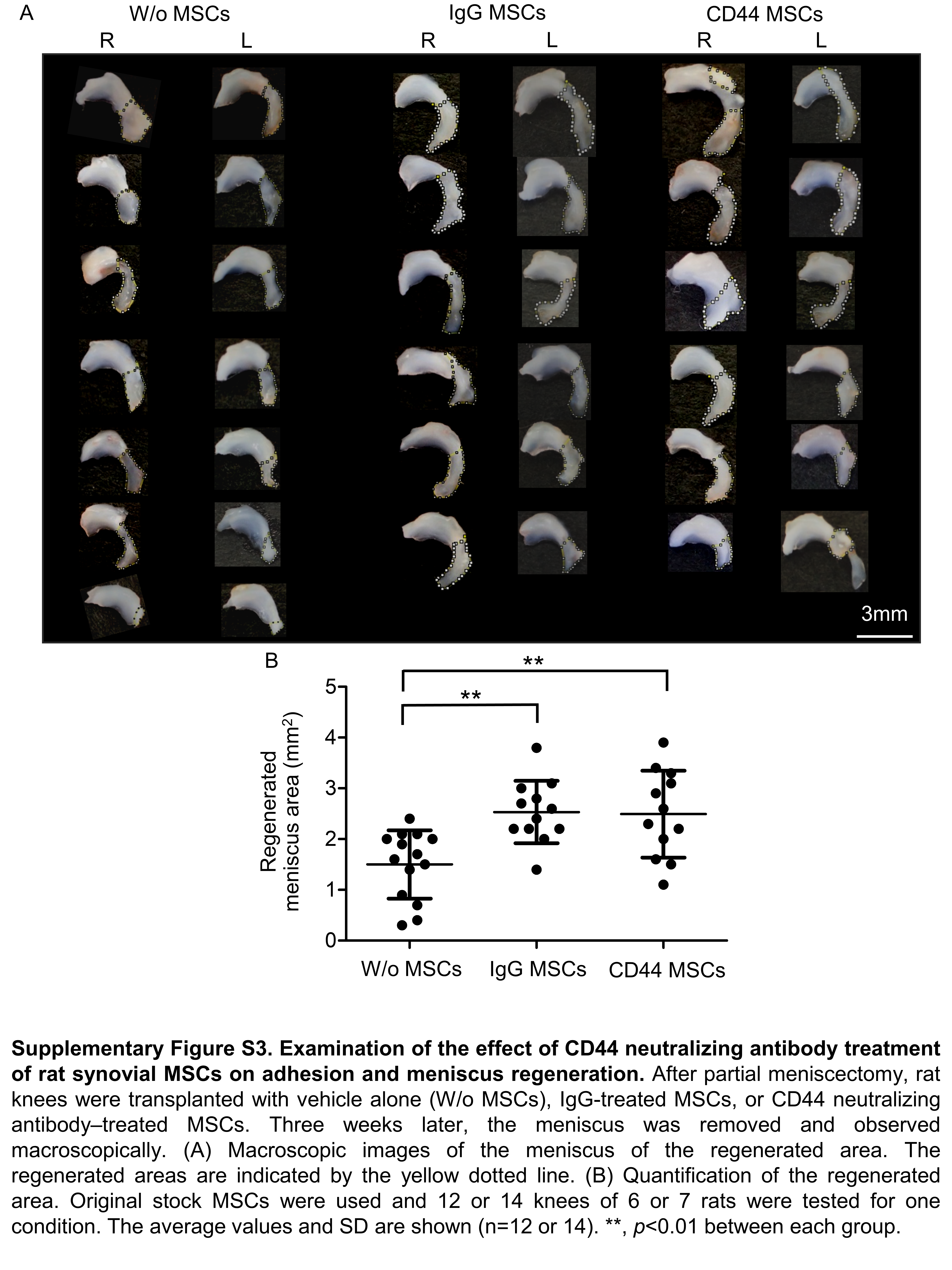

Supplement: Supplementary file 5 — Supplementary Information 5. [file 41598_2022_18476_MOESM5_ESM.tif]

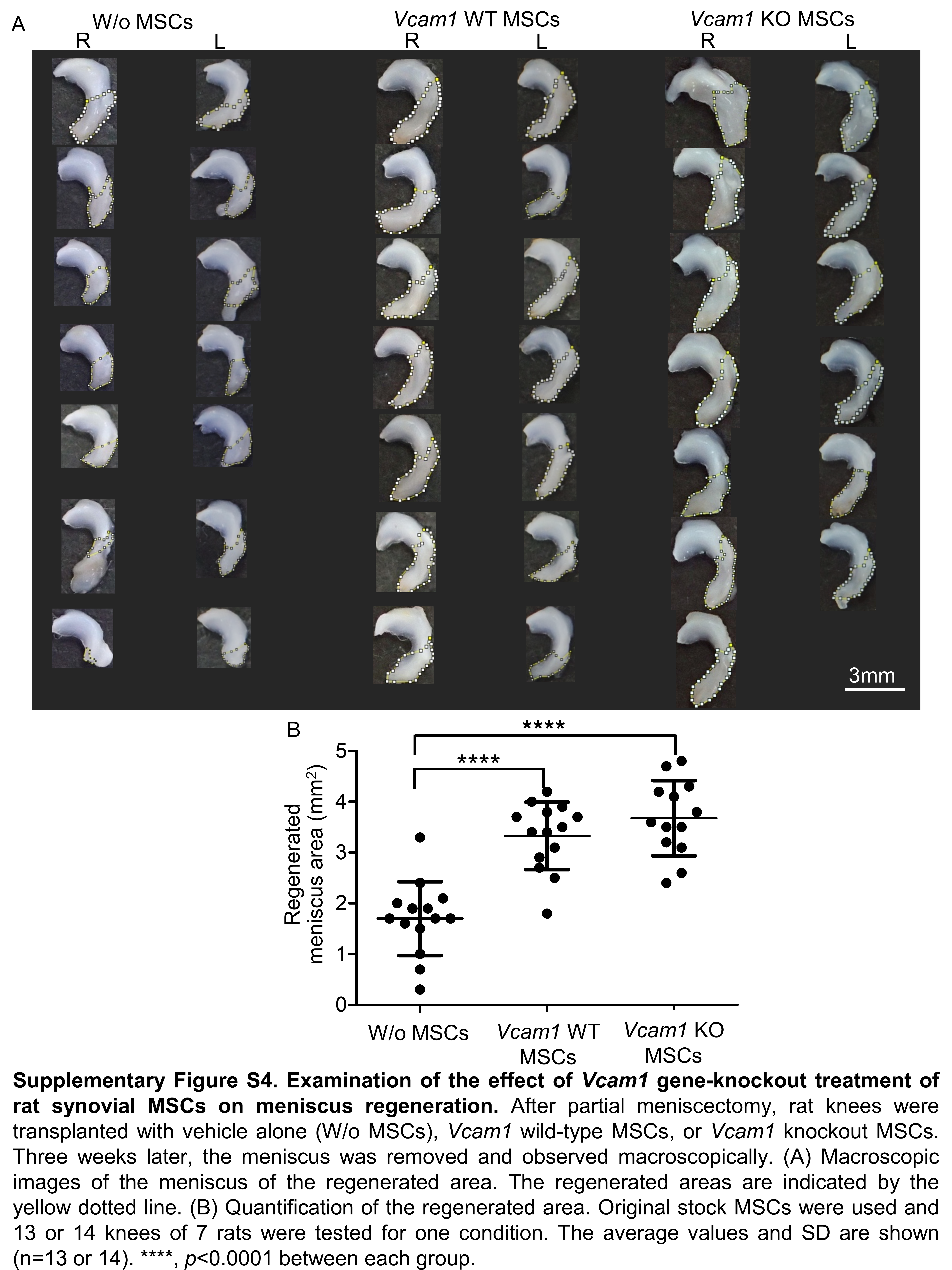

Supplement: Supplementary file 6 — Supplementary Information 6. [file 41598_2022_18476_MOESM6_ESM.tif]

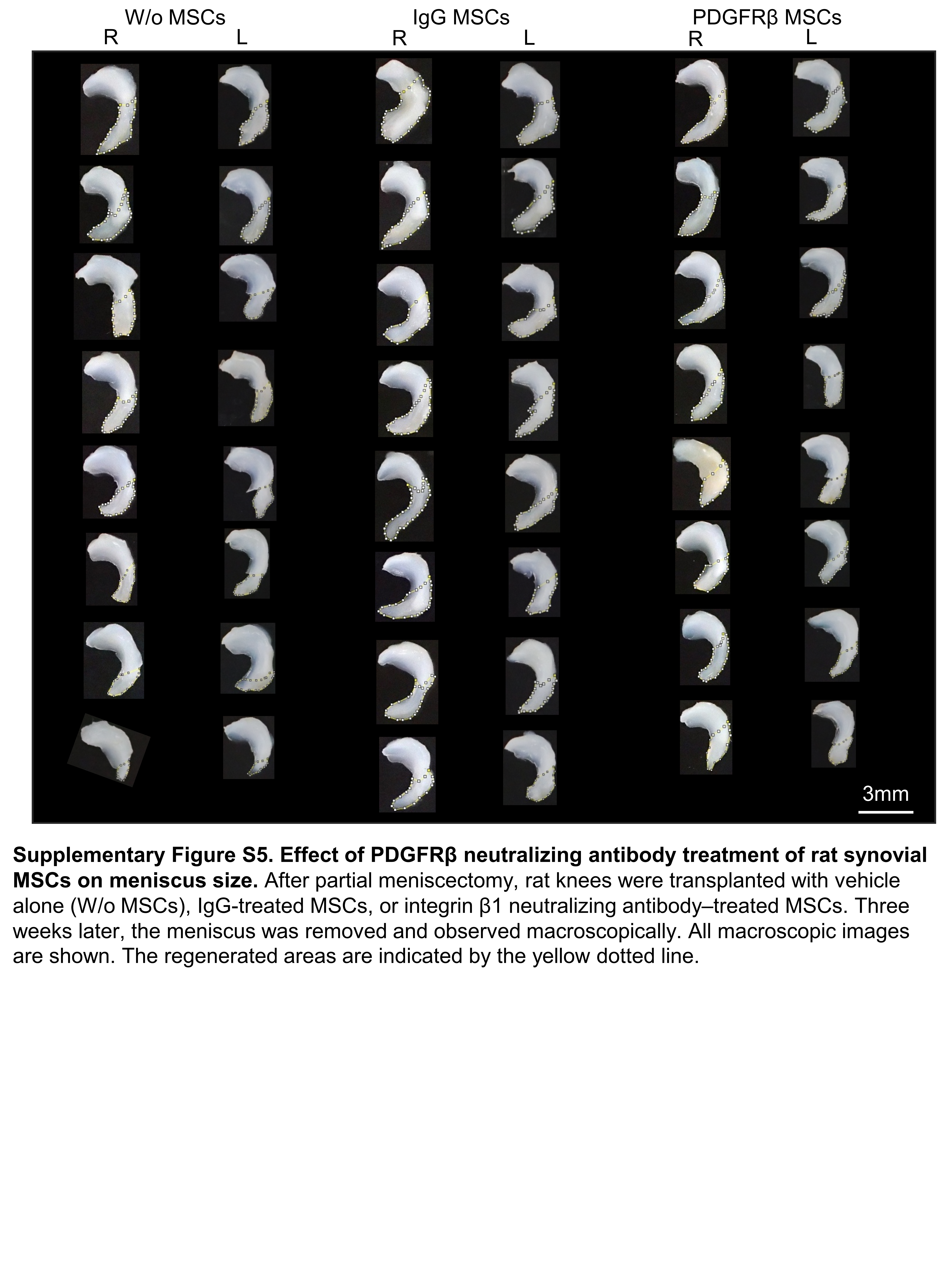

Supplement: Supplementary file 7 — Supplementary Information 7. [file 41598_2022_18476_MOESM7_ESM.tif]

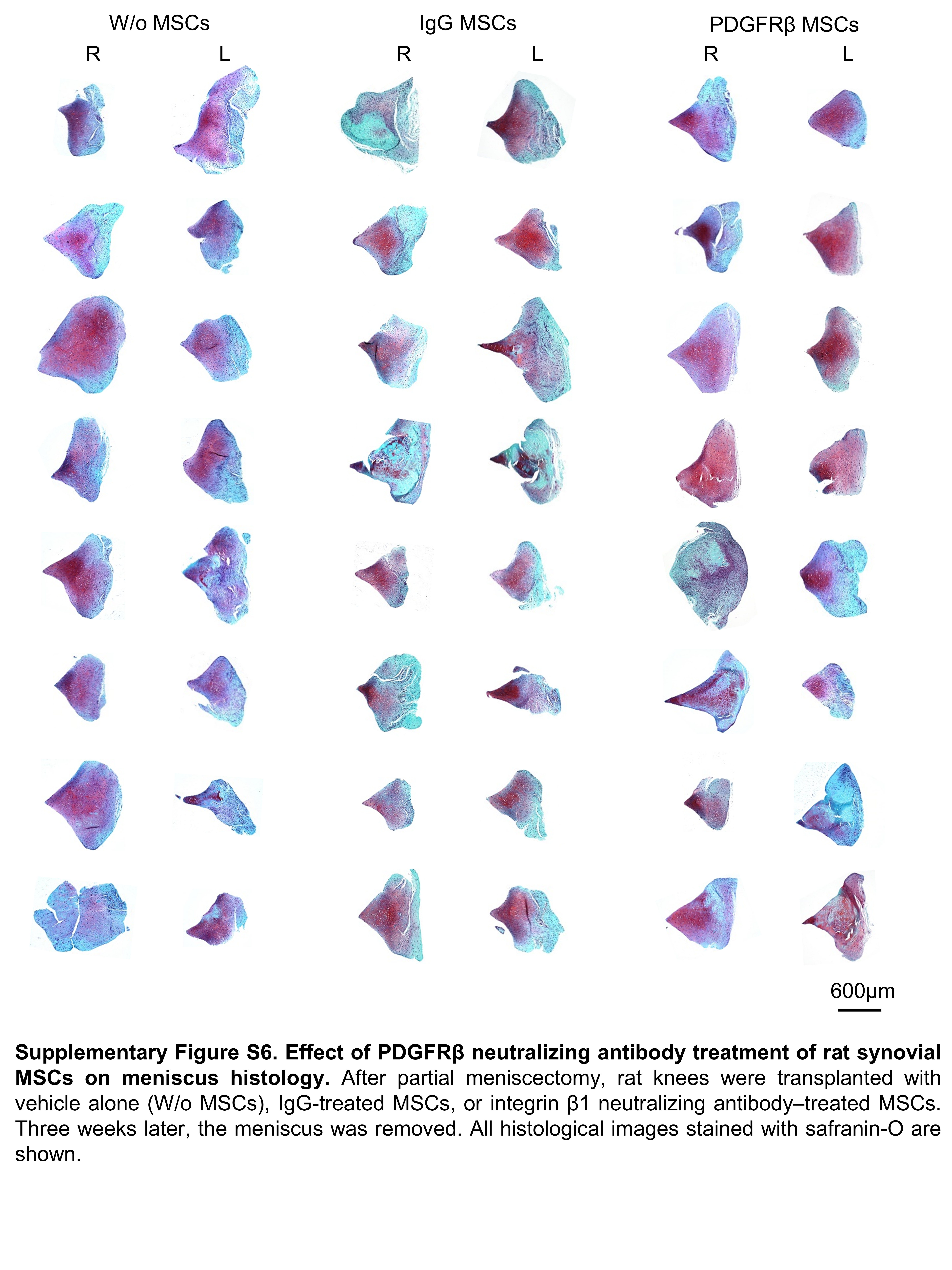

Supplement: Supplementary file 8 — Supplementary Information 8. [file 41598_2022_18476_MOESM8_ESM.tif]

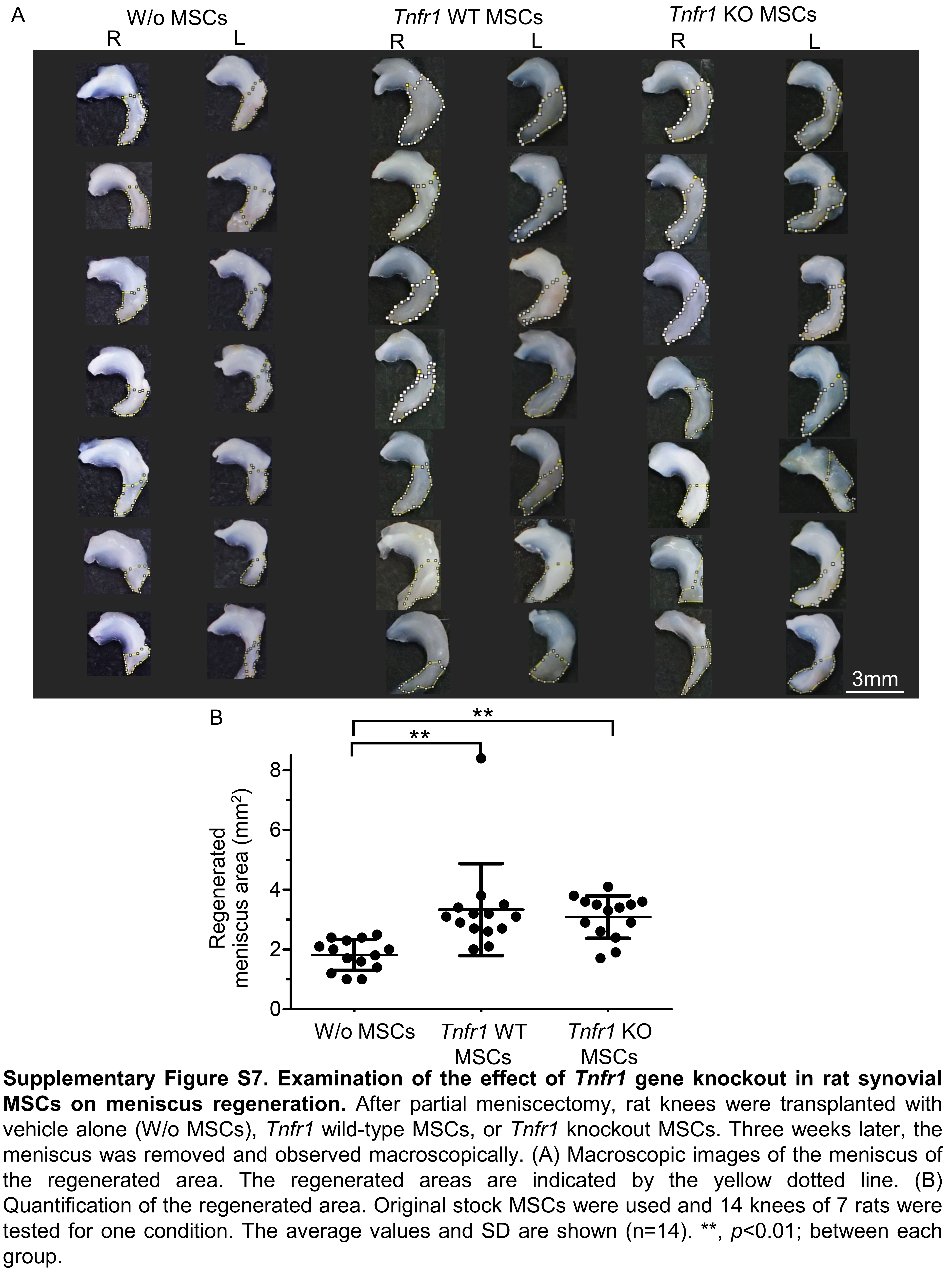

Supplement: Supplementary file 9 — Supplementary Information 9. [file 41598_2022_18476_MOESM9_ESM.tif]

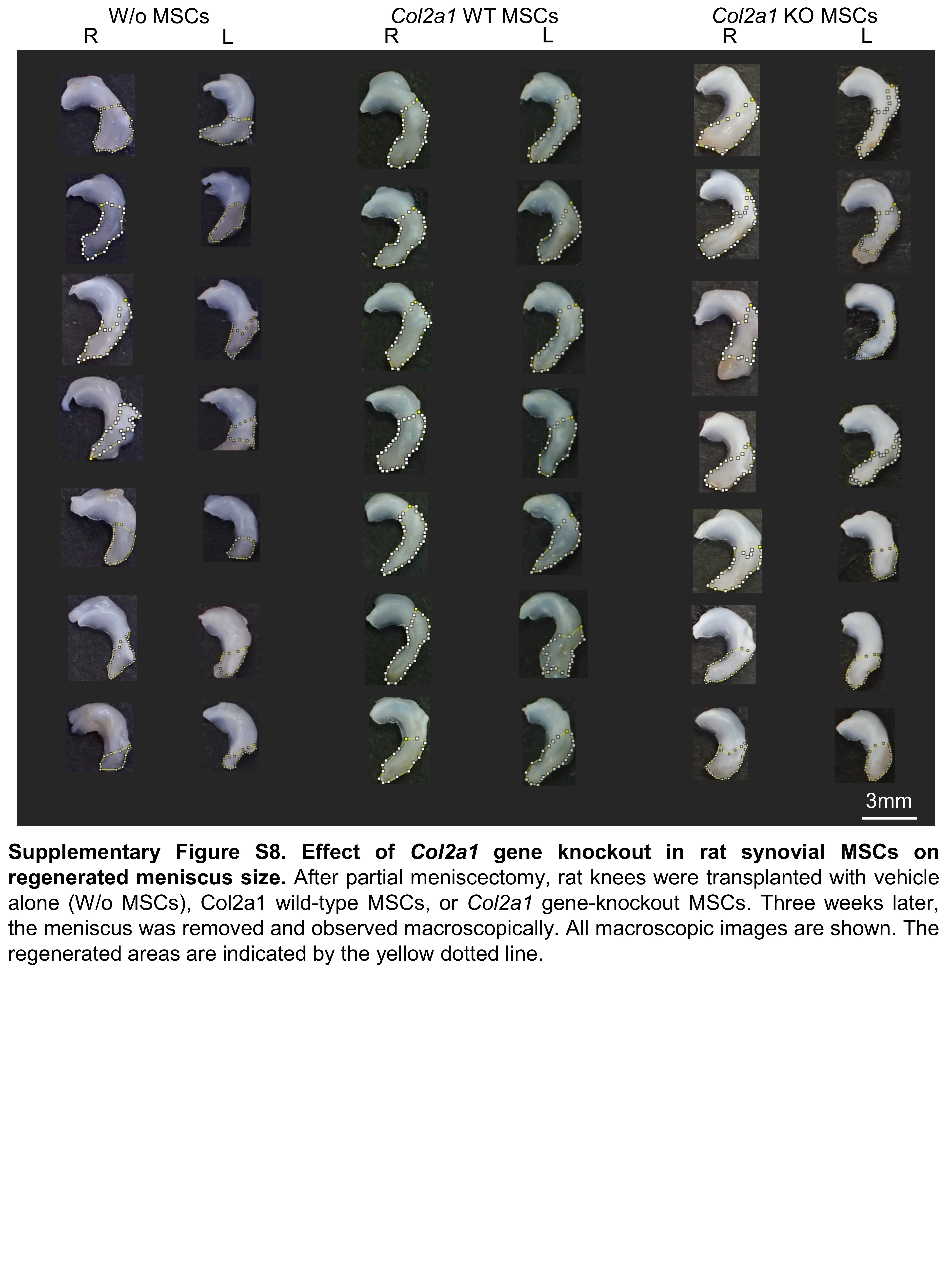

Supplement: Supplementary file 10 — Supplementary Information 10. [file 41598_2022_18476_MOESM10_ESM.tif]

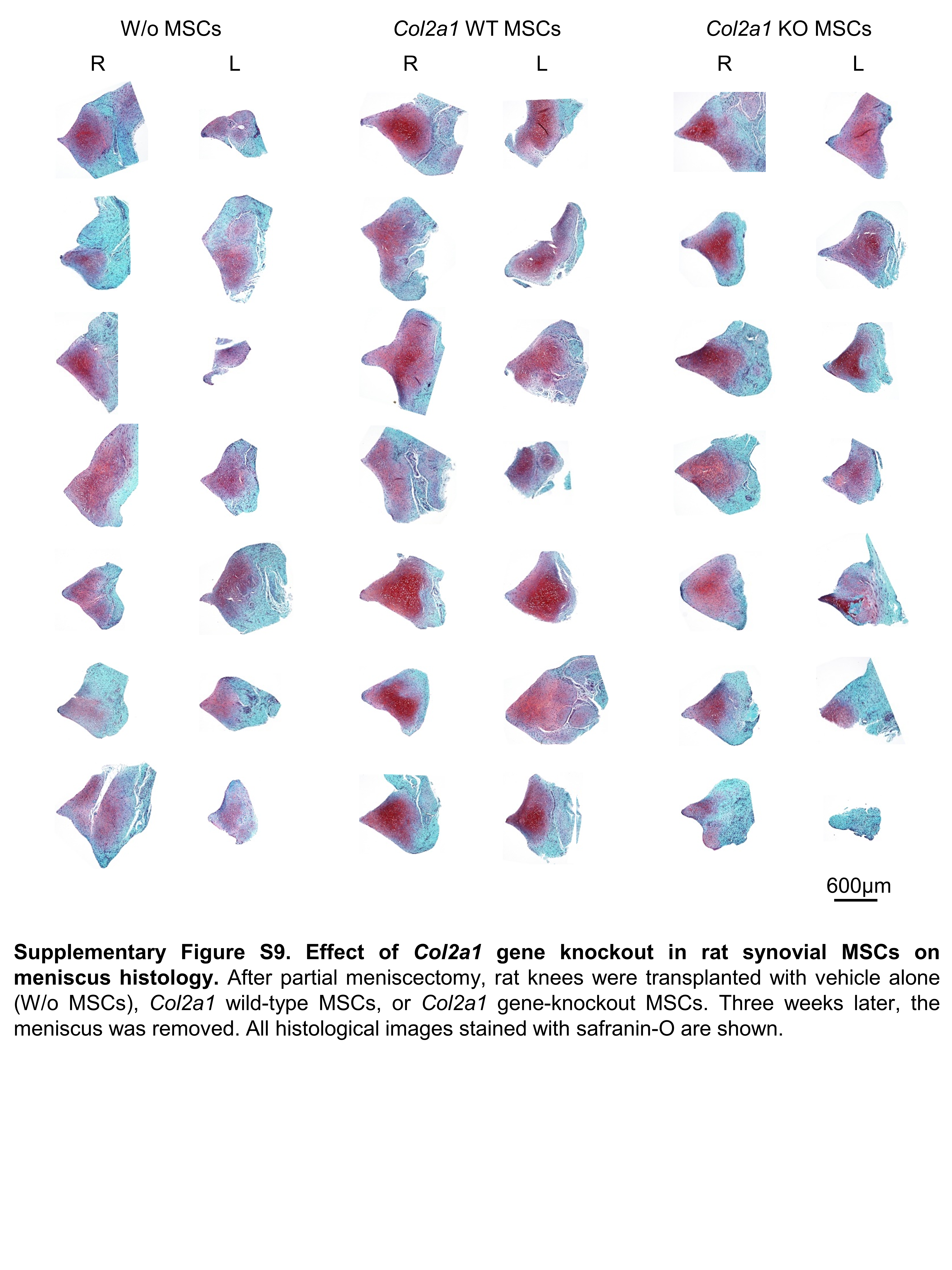

Supplement: Supplementary file 11 — Supplementary Information 11. [file 41598_2022_18476_MOESM11_ESM.tif]
